# Supplementary material for: Patterns of sedentary behaviour in adults with acute insomnia derived from actigraphy data
Source: PLoS One. 2023 Sep 21;18(9):e0291095. doi: 10.1371/journal.pone.0291095 (PMC10513233; doi:10.1371/journal.pone.0291095)
Supplement: Attachment — Submitted filename: Response to Reviewers.docx [file pone.0291095.s002.docx]

Dear Sir/Madam,

We would like to thank the editor and the reviewers for their valuable time, comments, questions and suggestions. All of them were taken into account, and the manuscript has been modified accordingly. We hope we have satisfied all queries and we believe the quality of the manuscript has significantly improved and is now of sufficient quality to warrant acceptance for publication. We will also put the analysis codes and data used in the paper to an open github online repository on final acceptance of the manuscript for publication.

Our detailed responses are given below in blue. The new changes made to the manuscript are highlighted in red.

Reviewer #1: Great work by authors. Keep it up. Please find my few suggestions in the attached PDF as comments.
here is an example
Page 7: Great efforts by the authors. Few suggestions from my side to make your article more understandable to the readers.
**Response/Action:**

We shifted all the table captions (table1- page 7, table2- page8, table3-page9, table4-page13, table5-page15) to the top of the tables.

If you add the study design to the title it will help us to understand lot more information clearly in your study.

**Response/Action:**

1.Following the suggestion, the title has been changed.

“Patterns of sedentary behaviour in adults with acute insomnia derived from actigraphy data”

2. We have rephrased the objectives of the study.

3. Moved text and table 1 (page 7) to the result section on page 7.

4. Table 2 - Abbreviations were explained in the caption on page 8 and captions are moved on the top of the table.

5. Table 3 - Abbreviations were explained in the caption on page 9 and captions are moved on the top of the table.

6. Table 3- sedentary bouts, total sedentary time and physical activity time is an event which are not normally distributed. To analyse these sedentary values we used the exponential distribution (discussed in materials and method section on page 5). Mean± lower/upper bound 95% confidence values, derived from the exponential distribution, are provided in Table 3 and table 4. Caption has been corrected to avoid confusion of these numbers.

7. Figures numbers have been mentioned and corrected in the manuscript(Figure 1- page 4 ,Figure 2- page 9, Figure 3- page 10, Figure 4- page 10, Figure 5- page 12, Figure 6- page 13, Figure 7- page 14)

8. Future suggestions were added on the page 16 at the end of the Discussion section.

Reviewer #2: Abstract:
Kindly elaborate more in detail on quantitative results of the present study findings

**Response/Action:**

Results in the Abstract are explained in more detail on page 1.

Introduction:
Overall, Introduction was well structured and covered the key topic area. However, you can add a note on the various methods of screening the sedentary behavior.

**Response/Action:**

As per suggestion we have added a paragraph about the screening methods of sedentary behaviour.

“There are several methods for measuring sedentary behaviour such as self-report questionnaires, direct observation, diaries or logs, and activity trackers. During specific periods, self-report questionnaires are used to estimate the amount of time participants spend sitting or engaged in sedentary activities, whereas direct observation involves trained observers watching individuals closely and recording their sedentary behaviour. Another method is to ask participants to keep a diary or log where they can record the start and end times of their sedentary periods and any relevant information. The use of activity trackers such as pedometers or wearable devices can provide objective data regarding sedentary behaviour. In addition to measuring movement, these devices can also estimate the amount of time spent sedentary based on periods in which a person is not active. It is important to note that each screening method has its strengths and limitations, and the selection should depend on the specific research or practical purpose. In order to provide a more comprehensive assessment of sedentary behaviour, it is beneficial to integrate multiple methods, or to incorporate objective measures along with self-report measures.”

Materials and methods:
The detailed structured methodology is lacking and its not well constructed and presented. Kindly rephrase the section with in detailed procedure.

**Response/Action:**

Several changes to Materials and Methods section and Results section were made to address the comment. The updated version includes a structured approach with subtitles for the different methods. Further details were added to selection and justification of the sample size. Table 1 and relevant text were moved to Results section.

EDITOR:
Please find the comments below:
1. Recommended to rewrite the abstract with sub-headings (structured)

**Response/Action:**

The Abstract has been rewritten in a structured way with sub-headings.

2. Provide clearly the selection criteria for both the groups.
3. The justification for the sample size must be provided.

**Response/Action:**

Selection criteria and justification of the sample size are provided on page 4.

4. Statistical methods are repeated. Please arrange the sections properly to avoid the repetitions.

**Response/Action:**

The statistical methods are rearranged (Sections Materials and Methods and Results) in order to avoid repetition.

5. The study findings are not supported with proper discussion. The main objective of the study is to determine the patterns of sedentary behavior. However, these findings were not discussed properly. It is recommended to rewrite the discussion analyzing the findings (week days, time of the day, ....) and causes for sedentary behavior patterns in participants with acute Insomnia.

**Response/Action:**

Study findings are explained in the Discussion section in paragraph 4 and 5 on page 15 and 16.

6. Discuss the reasons for sedentary behavior among the younger adults mentioning the other factors influencing the physical activity.

**Response/Action:**

Some possible reasons for sedentary behaviour are given in paragraph 1 of Discussion section on page 15. However, as the dataset had only actigraphy recordings and did not contain any information about the daily physical activities, it was not possible to make robust conclusions about the reasons for the sedentary behaviour based entirely on the actigraphy recordings.
