# Supplementary material for: Patterns of sedentary behaviour in adults with acute insomnia derived from actigraphy data
Source: PLoS One. 2023 Sep 21;18(9):e0291095. doi: 10.1371/journal.pone.0291095 (PMC10513233; doi:10.1371/journal.pone.0291095)
Supplement: Attachment — Submitted filename: Response to Reviewers.docx [file pone.0291095.s003.docx]

Dear Sir/Madam,

We would like to thank the editor and the reviewers for their valuable time, comments, questions and suggestions. All of these comments were taken into account, and the manuscript has been revised accordingly. We hope we have satisfied all queries and we believe the quality of the manuscript has significantly improved and is now of sufficient quality to warrant acceptance for publication.

Our detailed responses are given below in blue. The new changes made to the manuscript are highlighted in red. Grammar and language corrections are not highlighted in red.

**Journal Requirements:**

Please review your reference list to ensure that it is complete and correct. If you have cited papers that have been retracted, please include the rationale for doing so in the manuscript text, or remove these references and replace them with relevant current references. Any changes to the reference list should be mentioned in the rebuttal letter that accompanies your revised manuscript. If you need to cite a retracted article, indicate the article’s retracted status in the References list and also include a citation and full reference for the retraction notice.

**Response/Action:**

We have reviewed all the references for the retracted articles. There are no retracted articles cited in the manuscript and the references are formatted according to the journal's requirements.

**Additional Editor Comments:**

Language correction is required for the manuscript. Please double-check the grammar. Please ensure that each sentence in the abstract begins with a capital letter.

**Response/Action:**

We have reviewed the manuscript for grammar errors and ensured that every sentence in the abstract starts with a capital letter. The figures comply with the journal's requirements.
